# Evaluation of a multicomponent child development intervention delivered through the government health system: a feasibility study

**DOI:** 10.1136/bmjgh-2024-018736

**Published:** 2025-07-20

**Authors:** Jesmin Sultana, Helen O Pitchik, Abul Kasham Shoab, Tarique Md. Nurul Huda, Rezaul Hasan, Fahmida Akter, Tania Jahir, Md Khobair Hossain, Jyoti Bhushan Das, Md Ruhul Amin, Farzana Yeasmin, Rizwana Khan, Jenna E Forsyth, Laura H Kwong, Jahangir Rashid, Sabina Ashrafee, Mahbubur Rahman, Malay K Mridha, Fahmida Tofail, Peter J Winch, Stephen P Luby, Lia C. H. Fernald

**Affiliations:** 1 Environmental Health and WASH, Health Systems and Population Studies Division, International Centre for Diarrhoeal Disease Research, Bangladesh (icddr,b), Dhaka, Bangladesh; 2 Division of Epidemiology, University of California Berkeley School of Public Health, Berkeley, California, USA; 3 Department of Public Health, Qassim University, Buraydah, Saudi Arabia; 4 Centre for Environment and Population Health, School of Medicine and Dentistry, Griffith University, Brisbane, Queensland, Australia; 5 University of South Carolina, Columbia, South Carolina, USA; 6 Nursing and Health Sciences, University of Galway, Galway, Ireland; 7 International Centre for Diarrhoeal Disease Research, Bangladesh (icddr,b), Dhaka, Bangladesh; 8 Stanford University, Stanford, California, USA; 9 Division of Environmental Health Sciences, University of California Berkeley School of Public Health, Berkeley, California, USA; 10 Community Based Health Care, Directorate General of Health Services, Ministry of Health and Family Welfare, Dhaka, Bangladesh; 11 National Newborn Health Program and Integrated Management of Childhood Illness, Directorate General of Health Services, Government of the People’s Republic of Bangladesh Ministry of Health and Family Welfare, Dhaka, Bangladesh; 12 Global Health and Migration Unit, Department of Women's and Children’s Health, Uppsala University, Uppsala, Sweden; 13 Center for Non-communicable Diseases and Nutrition, BRAC James P Grant School of Public Health, BRAC University, Dhaka, Bangladesh; 14 Nutrition Research Division, International Centre for Diarrhoeal Disease Research, Bangladesh (icddr,b), Dhaka, Bangladesh; 15 Johns Hopkins Bloomberg School of Public Health, Baltimore, Maryland, USA; 16 Division of Community Health Sciences, School of Public Health, University of California Berkeley School of Public Health, Berkeley, California, USA

**Keywords:** Global Health, Child health, Epidemiology, Intervention study, Nutrition

## Abstract

**Introduction:**

Small efficacy trials have demonstrated that multicomponent interventions can improve early child development. We evaluated the large-scale delivery of a multicomponent intervention delivered by government health workers throughout a rural subdistrict in northwestern Bangladesh.

**Methods:**

We evaluated a group-based, multicomponent intervention with a curriculum covering responsive parenting, caregivers’ mental health, lead exposure prevention strategies at the household level, water, sanitation, hygiene and nutrition. Group sessions were held throughout a rural subdistrict of Bangladesh (August 2019–March 2020). A longitudinal sample of caregivers (n=517) of children 6–24 months was assessed at baseline and endline (primary cohort), and 1179 additional caregivers were assessed only at endline (supplementary cross-sectional). Outcomes were the variety of child play activities and materials, number of books, caregiver depressive symptoms and nutrition and lead knowledge. For primary analyses, we used difference-in-difference.

**Results:**

Over half (n=276, 53%) of the cohort participants attended any of the 16 intervention sessions and of these, 83% (228) attended 2+. Caregivers attending 2+ sessions, compared with ≤1 session, had more play materials (adjusted mean difference: 0.58; 95% CI: 0.30, 0.85) and were more likely to have any children’s books (adjusted prevalence difference (aPD): 0.26; 95% CI: 0.18, 0.34), to have heard of lead (aPD: 0.13; 95% CI: 0.07, 0.19) or to know how to avoid harm from lead (unadjusted PD: 0.13; 95% CI: 0.08, 0.17). These findings were similar to those from the supplementary cross-sectional analysis. There were no differences in caregiver depressive symptoms in either analysis. More child play activities and nutrition knowledge were associated with attendance in the cross-sectional sample.

**Conclusions:**

A multicomponent child development intervention delivered by government health workers increased the presence of children’s toys and books and caregiver knowledge of lead in families who attended two or more sessions. Further adaptation and alternative delivery methods are likely to improve the reach and the breadth of impacts.

**Trial registration number:**

NCT04111016.

**WHAT IS ALREADY KNOWN ON THIS TOPIC**

- Multicomponent, child development interventions delivered at a small scale by delivery agents hired specifically for the project can improve child development outcomes in low-resource settings.

**WHAT THIS STUDY ADDS**

- A multicomponent group-based intervention implemented by the government health system can (1) initially reach around half of the target population and (2) improve the availability of play materials at home, number of children’s books, and knowledge of lead, but not nutrition knowledge, variety of play activities or caregiver depressive symptoms, among caregivers who attended at least two group sessions.

**HOW THIS STUDY MIGHT AFFECT RESEARCH, PRACTICE OR POLICY**

- Early child development support programmes have the potential to be scaled through existing government health systems; however, further adaptation is needed to reach and sustain engagement from caregivers.

## Introduction

 Hundreds of millions of children under 5 living in low- and middle-income countries (LMICs) are at risk for poor motor, cognitive and socioemotional development.^1^ Multiple early life exposures contribute to poor early child development, including a lack of early stimulation, inadequate nutrition, poor water, sanitation and hygiene (WASH) and lead exposure.^1 2^ Early motor, cognitive and socioemotional deficits can lead to poorer academic attainment^3^ and literacy,^4^ and fewer economic opportunities, contributing to intergenerational cycles of poverty and poor health.^5 6^

For these reasons, Sustainable Development Goal 4.2 is to ensure that children get quality early care and preprimary education,^7^ ideally through responsive caregiving, integration of caregiving and nutrition interventions, and a supportive environment for maternal mental health.^8^ Parenting interventions have demonstrated consistent effectiveness in terms of promoting early child development across many cultures and contexts.^9^,^12^

However, many of the evaluated interventions are either delivered at a small scale, or through study-specific mechanisms that are not feasible at scale, and these interventions do not necessarily translate to impacts when implemented at scale.^13^,^15^ Small-scale interventions often depend on individual relationships and are delivered by non-governmental entities and may be limited in scope.^13 14^ Programmes delivered through government health systems have a greater potential for scale as government health systems are designed to reach the entire population living within a given catchment area. These broader approaches, however, require political engagement, community support and institution building,^14 16^ and need increased organisational capacity and strategic partnerships with governmental and non-governmental organisations.^13 14^ A review of selected scaled-up early child development interventions found that the majority of the scaled interventions were delivered through the health system.^17^ However, there are concerns that adding to an overburdened and underperforming health system may not result in successful implementation.^15^ Several studies have evaluated the effects of large-scale home visiting interventions delivered through systems that have the potential to scale up to entire catchment areas and have produced mixed results; some show no effects on child outcomes,^18^,^20^ while others have shown benefits.^21^,^23^

When designing interventions with the goal of scaling up in mind, intervention cost and cost-effectiveness are critical components.^17^ Interventions delivered to larger groups of caregivers have the potential to be less costly than home visits or intervention sessions delivered to small groups, and previous work from small-scale evaluations demonstrates evidence of their potential to improve child development outcomes.^9 10 24^ In Bangladesh, three studies that evaluated small (two to four caregivers) group sessions have improved child development outcomes when delivered through community health clinics,^25^,^27^ but larger group sessions at these clinics have not been tested. Further, all three of these studies restricted intervention enrolment to 25 or fewer caregivers per community clinic, thus integrating delivery through existing government services, but not delivering the intervention at scale in these locations.^25 26^

The goal of this study was to evaluate a multicomponent child development intervention delivered by government health workers to groups of caregivers and young children throughout Chatmohar, a rural subdistrict in northwestern Bangladesh.

## Methods

### Study setting

The study was conducted in the Chatmohar subdistrict of Pabna district, Bangladesh; the population size was 291 121 in 2011 and the main source of income for residents was agriculture.^28^ Chatmohar is located north-west of Dhaka and is composed of 11 rural unions (smallest administrative unit of Bangladesh) and one urban municipality. At the 2011 census, 46% of the residents over 7 years old were literate, 96% were Muslim, 83% lived in houses made of mud, bamboo, or corrugated iron (‘katcha’), and 96% of households owned the house they lived in.^28^

### Study design and participants

**Figure 1:**
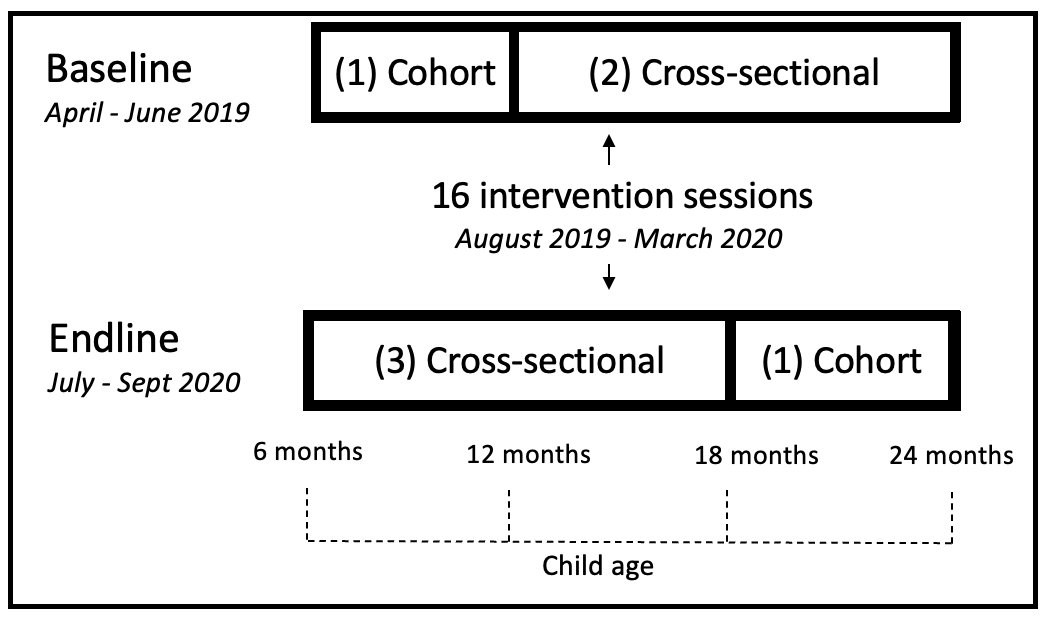
Visual representation of the study design and intervention timeline.

The study design was a pre–post cross-sectional age-matched design with a nested longitudinal cohort (figure 1). The design was meant to enable a primary analysis within the longitudinal cohort as well as additional analyses with the cross-sectional samples to triangulate evidence within the observational research design. The baseline assessment was conducted in person at the home of primary caregivers of children aged 6–24 months old who were selected through multistage population proportional sampling (online supplemental box 1). The endline assessment included a follow-up of caregivers of children 6–12 months at the baseline assessment (the longitudinal cohort, around 18–24 months old at endline), and an additional cross-sectional sample of newly recruited children 6–18 months (figure 1). The cross-sectional endline sample was recruited from households identified during the baseline household screening and conducted on the phone, due to the COVID-19 pandemic. At endline, caregivers who refused, migrated or did not respond to the phone call after multiple attempts were replaced by the next household on the list. We excluded children who were hearing, vision or speech impaired, and at baseline, we only included caregivers who planned to reside in the study area for at least 1 year.

### Intervention

The Research on Integration of Nutrition, Early childhood development and WASH through the Government health system (RINEW-G) intervention was developed by adapting a previously evaluated group-based multicomponent intervention for delivery through the government health system.^29^,^31^ Healthcare workers invited all primary caregivers of children under 12 months of age and pregnant people living in Chatmohar subdistrict (n=7718) to attend intervention sessions at the nearest primary healthcare facility and distributed a health card to keep track of session attendance. Caregivers of children over 12 months were allowed to attend intervention sessions but were not explicitly recruited. The intervention was implemented from August 2019 to March 2020. The intervention was designed to last for 12 months, but activities were suspended after 8 months due to the COVID-19 pandemic.

The intervention was delivered to groups of pregnant women or caregivers at primary healthcare facilities. Participants at each healthcare facility were formed into 10–15 initial groups of 10–15 participants. Group size was selected based on the space available at community health centres as well as initial pilot sessions to determine the feasible size for engaging delivery. Participants who lived near each other were grouped to enhance group cohesion. Pregnant women attended group meetings during their scheduled monthly antenatal care (ANC) visits, and sessions for caregivers were held twice a month. A total of 16 unique group sessions were offered for caregiver–child dyads and 4 were offered for pregnant women (which repeated over the session duration, details in online supplemental table 1). Since each facility had 10–15 participant groups, facilitators organised each of the 16 sessions 10–15 times to ensure all participants had the opportunity to receive the full intervention.

Intervention sessions included behavioural recommendations for early childhood stimulation adapted from the Reach-Up curriculum,^32^ maternal and child nutrition adapted from the government of Bangladesh guidelines and previous nutrition interventions in Bangladesh,^33 34^ best practices relating to WASH adapted from WASH-Benefits trial,^34^ maternal mental health adapted from the Thinking Health programme,^35^ and lead exposure prevention adapted from a previous intervention in Bangladesh.^31^ Lead exposure, though an established cause of poor child development in Bangladesh, is not usually included in early child development interventions, despite lead being a common exposure for children in LMICs^36^ with irreversible impacts on children’s development.^37 38^ This is likely due to unknown sources of exposure.^39^,^41^ The lead component of this intervention was developed and piloted in the previous small-scale implementation of the intervention.^31^

Study participants received age-appropriate toys made from recycled materials, simple picture books, a food group poster, micronutrient powder for children 6–24 months old, iron and folic acid tablets for pregnant and lactating women with a child <3 months, and kits for Kangaroo Mother Care for low-birth-weight newborns (online supplemental table 1). Sessions for caregiver–child dyads alternated between providing new messages and a focus on reviewing materials and practising activities with young children.

71 government healthcare workers who were already working in one of the 45 community healthcare facilities in Chatmohar were trained to facilitate group sessions. Among the 71 healthcare workers, 60% were female and 40% male, with a mean age of 35.2 years (SD: 9.4). Healthcare workers had an average of 14.6 years (SD: 2.3) of education and 11.8 years of work experience (SD: 9.0). Other duties of healthcare workers depended on their title and included delivering family planning, antenatal and postnatal care services as well as basic healthcare services and nutrition education (online supplemental table 1). They attended an initial 6-day training session meant to provide facilitators with both technical and practical training. The sessions comprised in-house facilitated discussion using audio-visual training materials, which included a lecture session followed by demonstration of play activities, training on tablet computers, role play and field testing with non-study households. The training was conducted by topic experts from the study team who were involved in developing the intervention curriculum. An additional 3-day training was conducted after 4 months to review the subsequent 4 months of intervention sessions. Session facilitators delivered sessions with the help of tablet computers, which guided them to provide age-specific intervention content, as well as toys, picture books and illustrative posters. The joint monitoring team, consisting of members from the icddr,b study team and government supervisors of the primary healthcare workers involved in the study, monitored session facilitators during at least one of each unique session delivered by each facilitator and provided supportive supervision for improving the session delivery quality. During the study, government supervisors incorporated facility visits and session supervision into their monthly work plan.

### Intervention and comparison groups

Primary caregivers who reported attending at least two (2+) intervention sessions were considered to be exposed to intervention sessions (‘intervention’ group), and those who reported attending one or no intervention session (≤1) were considered to be unexposed to intervention sessions (‘comparison’ group). As outlined in our preanalysis plan (https://osf.io/b7p2c/?view_only=99e2d5c9e6b449efaddb9cf1c7080b44), we included those who attended one session in the comparison group because the first intervention session was an overview session and did not include many concrete behavioural recommendations or practices. We included individuals who attended 2+ sessions in the intervention group because these individuals were guaranteed to attend at least one intervention session with in-depth session contents and practice engaging with their children (e.g., they did not only attend the first intro session).

### Assessments

The assessments included sociodemographic information in addition to measures of each of the outcomes of interest. We assessed the stimulating home care environment using the Family Care Indicators, which have been used previously in Bangladesh.^42^ The stimulating caregiving practices subscale has questions about the engagement of the child’s parents, or other adults (over 15 years old) in six activities that can promote cognitive stimulation with the child over the previous 3 days. The primary analysis used the participation reported for the child’s mother (range 0–6) because the mother was most often interviewed and assumed to give the most accurate report. We conducted two analyses with this outcome, one with a continuous measure (0–6) and one with a binary measure indicating if the child received four or more stimulation activities, consistent with analyses done previously.^43^ The variety of play materials subscale includes data regarding various types of play materials the child used to play during the previous 30 days (range 0–6). The number of children’s books in the home was collected through self-report, corroborated by observation at baseline. Sources of books were recorded as bought, received from government health workers or from elsewhere.

Assessment of caregiver depressive symptoms was conducted with the Centre for Epidemiologic Studies Depression scale (CES-D), which includes 20 questions about the number of days in the previous week that the caregiver experienced each symptom. Scores range from 0 to 60, where a higher value indicates more frequent depressive symptoms.^44^ The CES-D has been used previously in Bangladesh.^45 46^ We examined the reliability of the CES-D scale in our study sample and found relatively high reliability at both time points (alpha=0.83 at baseline and 0.84 at endline for the cohort sample).

Caregiver knowledge about nutrition was assessed through six questions about maternal and child nutrition, with the number of correct answers summed (final score range 0–6, online supplemental table 2). Caregiver knowledge of lead was assessed through two questions analysed separately. Any knowledge of lead was assessed through one question with a binary response ‘Do you know what lead is?’. Knowledge of ways to avoid harm from lead was assessed with participants naming an appropriate method to avoid harm from lead.

Graduate-level (≥15 years of education) data collectors were trained through facilitated discussion, role-play and field testing. The training sessions were conducted by study team members. The baseline training was a 3-week in-person training, and the endline training session was a 2-week training consisting of both online and in-person sessions. A field supervisor shadowed at least one data collector daily to assess the data collection quality and provided supportive supervision throughout the data collection period.

### Statistical analysis

Primary analyses were prespecified in an analysis plan developed before data analysis (https://osf.io/b7p2c/?view_only=99e2d5c9e6b449efaddb9cf1c7080b44). For the primary analysis to examine the association between exposure to intervention sessions and the outcomes of interest in the longitudinal cohort, we used difference-in-difference. We compared differences in outcomes from baseline and endline between participants who were exposed to 2+ intervention sessions and those who were exposed to ≤1 intervention session. We used generalised estimating equations with clustered standard errors at the village level, controlling for theoretical confounders. The initial sample size was powered to detect a 0.40 SD difference in continuous outcomes between those who did and did not attend intervention sessions (80% power, alpha of 0.05).

As a secondary analysis meant to triangulate findings in our primary longitudinal analysis, we compared differences in outcomes for those exposed to 2+ intervention sessions and ≤1 intervention session among the cross-sectional endline sample. Methods specific to the secondary analysis are presented in online supplemental box 2.

To note, we had anticipated a third comparison between the full baseline and endline age-matched samples, however, given potential bias from differences in sampling methods due to the COVID-19 pandemic, we did not conduct this third comparison.

In both primary and secondary analysis, we adjusted for multiple theoretical confounders including child age (in months), child sex, maternal and paternal education (completed primary education), household income (in tertiles), housing materials (presence of concrete floors and brick walls compared with soil or tin), 4+ANC visits, number of children <15 years in the household (categorised into 1, 2 and ≥3 children), caregiver control over assets (spending their own money independently), and caregiver involvement in household decision-making (participation in at least 5 of 7 decisions). For the children’s books, knowledge of lead and ways to avoid harms from lead outcomes, we had low prevalence at baseline and reduced the confounder set or changed the model type to achieve model convergence (noted in the results tables where applicable, for example, for caregiver knowledge of ways to avoid harm from lead unadjusted estimates from a linear probability model are presented for the longitudinal cohort results due to lack of convergence).

#### Sensitivity analyses

We conducted sensitivity analysis for different session attendance cut-offs, including (1) categorical exposure of 0, 1–2, 3–5 or 6+ sessions attended and binary comparisons of (2) 2+ vs 0 sessions and (3) 1+ vs 0 sessions. We also examined an alternative play activity score which captured play activities with any adult from the household. We additionally conducted subgroup analyses by experiences of financial loss and food insecurity during the COVID-19 pandemic (by including an interaction term). Finally, we repeated the primary analyses using inverse probability of loss to follow-up weights. The sensitivity analyses of binary comparison of 1+ vs 0 and alternative play activity score were not included in our pre-analysis plan. We added these analyses to further validate our primary analysis findings. We had initially proposed to create propensity scores for attending 2+ intervention sessions and trimming the sample if there was a lack of overlap. Limited lack of overlap was found, and so we did not conduct analyses with a trimmed sample.

## Results

### Characteristics of the study sample

**Figure 2:**
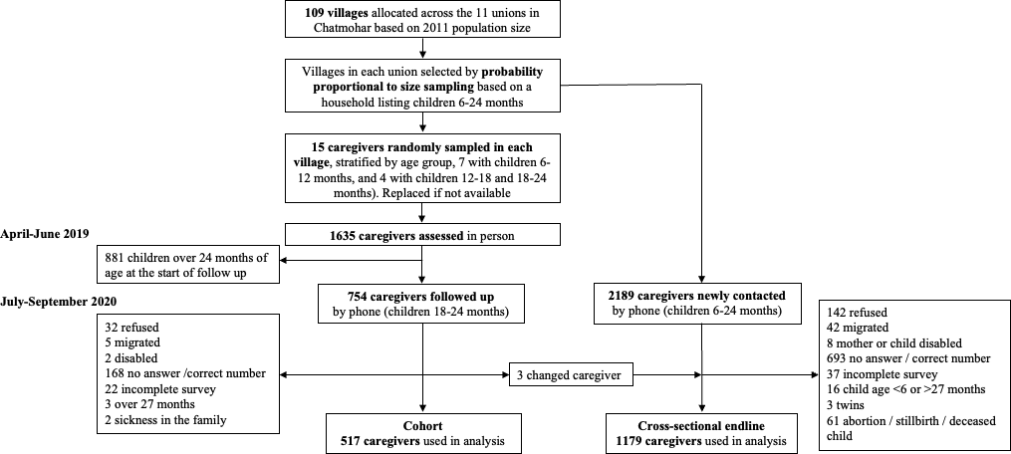
Study sample flow chart.

At baseline, 1635 caregivers of children aged 6–24 months were assessed (April–June 2019). Of these, 754 caregivers had children under 14 months and were followed up as part of the longitudinal cohort. At endline (July–September 2020), 523 participants were reassessed from the longitudinal cohort sample (69% of those with children <14 months at baseline), and an additional 1176 participants were newly recruited for the endline cross-sectional sample. Six participants from the cohort sample were excluded from the cohort analysis: three were >27 months at the time of assessment and three children changed primary caregivers (those who changed caregivers were included in the cross-sectional endline sample). In the cohort sample, the primary reasons for the loss to follow-up were no answer to the phone call or incorrect phone number (n=168) and participant refusal (n=32) (figure 2). Most of the sociodemographic characteristics among participants who were lost to follow-up were similar to those who were assessed at the endline sample, except those lost to follow-up had fathers who were less likely to have completed primary education and were more likely to be in the lowest tertile of income (online supplemental table 3).

#### Primary cohort sample

At baseline, the mean age of primary caregivers in the cohort (516 mothers and 1 grandmother) was 25 years, and over 80% had received at least 5 years of formal education. At baseline, children’s ages were similar in both the intervention and comparison groups (mean around 8.5 months, overall range 4.9–13.8 months). Though we intended to enrol children 6–12 months at baseline, some caregivers initially reported incorrect birthdates, and the actual age of enrolment extended outside of the intended range. Around half of the children were female. Household characteristics including household size, number of children under 15, cemented floor and brick walls were similar in both study groups at baseline. During their last pregnancy, 92% (n=209) of mothers in the intervention group and 85% (n=247) of mothers in the comparison group attended at least one ANC session (table 1).

**Table 1:** Demographic characteristics of the cohort sample at baseline stratified by session attendance

	Session attendance	
	2+ (n=228)	≤1 (n=289)
Primary caregiver characteristics	**% (n) or mean±SD**	
Age (in years)	25±5.2	25±5.4
Completed primary education (5+ years)	80% (183)	81% (233)
Currently pregnant	1.8% (4)	0.69% (2)
Muslim	99% (226)	97% (281)
Father characteristics		
Father completed primary education (5+ years)	59% (132)*	65% (187)†
Child characteristics		
Age (in months)	8.3±1.8	8.7±1.8
<6 months	8.8% (20)	4.8% (14)
6 to <14 months	91% (208)	95% (275)
Female	46% (106)	54% (155)
Household characteristics		
Household size	5.2±1.7	5.3±1.9
Children <15 years old under care of primary caregiver	1.9±0.75	1.8±0.84
Has cement floor	18% (41)	19% (56)
Has brick walls	20% (45)	26% (76)
Antenatal care visits during pregnancy		
Attended at least one visit	92% (209)	85% (247)
Attended at least four visits	35% (80)	37% (106)
Household income tertile‡		
Low	51% (117)	49% (142)
Medium	25% (58)	21% (62)
High	23% (53)	29% (85)
Caregiver can spend money independently	59% (134)	55% (158)
Caregiver involved in decision-making process (5+ activities out of 7)	57% (130)	54% (157)

When missing data are present, n’s are noted in footnotes.

* n=225, three observations missing.

† n=287, two observations missing.

‡ Tertile is based on the entire baseline sample including those not followed in the cohort (n=1635).

#### Secondary endline cross-sectional sample

Among the 1179 participants in the endline cross-sectional sample, the ages of children were similar in the intervention (18.2 months, SD 5.6) and comparison (16.6 months, SD 5.5) groups (range 7.2–26.9 months). Comparison group children were more likely to have a cemented floor (24% vs 17%), and brick walls (30% vs 22%). There was no difference observed in terms of other measured characteristics between the intervention and comparison groups (online supplemental table 4).

### Session attendance

In the cohort sample, 53% (n=276) of participants attended any of the 16 offered intervention sessions. Of those that attended any sessions, 17% (n=48) attended only one session, and 83% (n=228) attended two or more sessions (average 2.6 sessions, SD 3.7, range 0–16, median: 1) (online supplemental table 5). Of those who attended two or more sessions, the mean sessions attended was 5.6 (median 4, IQR 3–7) or 35% of the 16 offered sessions.

In the cross-sectional endline sample, 40% (n=477) of participants attended any intervention session, and of those, 71% (n=337) attended 2+ sessions (online supplemental table 5).

### Differences across groups

#### Primary difference-in-difference analysis with cohort sample

Participants who attended 2+ sessions had more play materials (adjusted mean difference (aMD): 0.58, 95% CI: 0.30, 0.85), were more likely to have 1+ children’s book (adjusted prevalence difference (aPD): 0.26, 95% CI: 0.18, 0.34) and were more likely to have heard of lead (aPD: 0.13, 95% CI: 0.07, 0.19) as well as knew more ways to avoid harm from lead (unadjusted PD: 0.13; 95% CI: 0.08, 0.17) compared with participants who had attended ≤1 session (table 2). The number of play activities children participated in with their primary caregiver and caregiver nutrition knowledge were positively associated with caregiver attendance at 2+ intervention sessions, and caregiver depressive symptoms were negatively associated, though CIs for these associations all crossed the null (table 2).

**Table 2:** Results from DIDs analyses in the longitudinal cohort (n=517)

Outcome	Baseline		Endline		Adjusted DID (95% CI)¶
	Session attendance				
	2+ (n=228)	≤1 (n=289)	2+ (n=228)	≤1 (n=289)	
	Mean±SD or % (n)				
CES-D score	13±8.9	13±8.6	13±9.0	13±9.4	−0.47 (−1.98, 1.03)
CES-D score above median (>12)*	46% (104)	43% (123)	40% (91)	41% (118)	−0.04 (−0.14, 0.06)
FCI play activities	2.5±1.4	2.5±1.6	3.9±1.5	3.7±1.6	0.23 (−0.11, 0.57)
FCI play materials	1.2±1.2	1.2±1.1	3.1±1.3	2.6±1.2	0.58 (0.30, 0.85)
FCI play activities (4+)	25% (57)	28% (80)	62% (142)	59% (171)	0.04 (−0.06, 0.15)
1+ children’s books†	4.8% (11)	2.4% (7)	75% (172)	47% (136)	0.26 (0.18, 0.34)
Caregiver nutrition knowledge	2.1±1.1	1.9±1.0	2.7±1.3	2.4±1.3	0.08 (−0.16, 0.31)
Caregiver has heard of lead‡	4.8% (11)	4.2% (12)	19% (43)	4.8% (14)	0.13 (0.07, 0.19)
Caregiver knowledge of ways to avoid harm from lead§	0% (0)	0.7% (2)	13% (30)	1% (3)	0.13 (0.08, 0.17)

Outcomes: CES-D, CES-D 20-question version (range 0–60, higher scores indicate more depressive symptoms); FCI play activities, FCI play activities subscale (range 0–6) is the total number of play activities that the primary caregiver participated in with the child in the preceding 3 days; FCI play materials, FCI play materials subscale (range 0–6) is the variety of play materials child played with within previous 30 days that were observed in the home; 1+ children’s books indicates the presence of any children’s books in the home; caregiver nutrition knowledge is a sum score of correct responses to six questions about maternal and child nutrition (0–6); caregiver has heard of lead is a binary outcome in response ‘Do you know what lead is?’; caregiver knowledge of ways to avoid harm from lead is a binary outcome that indicates at least one correct response to a question about knowledge of ways to avoid harm from lead.

Adjusted DID: (mean differences for continuous outcomes and prevalence differences for binary outcomes) from a generalised estimating equation model adjusted for child age, child sex, maternal and paternal education (completed 5+ years of education), household income (categorical), 4+ antenatal care visits, control over assets, number of children <15 years old (categorical), housing materials (concrete walls and floors) and maternal involvement in the decision-making process.

* Baseline median CES-D score is 12.

† Model not adjusted for father’s education due to lack of convergence.

‡ Model only adjusted for child age, mother’s education, 4+ antenatal care visits due to lack of convergence.

§ Unadjusted estimate is presented from a linear probability model with robust SEs due to lack of convergence with adjusted GEE model.

¶ n=516 for adjusted analyses that adjust for paternal education (1 missing observation)

CES-D, Centre for Epidemiologic Studies Depression scale; DID, difference-in-difference; FCI, family care indicator; GEE, generalised estimating equation.

#### Secondary analysis with the cross-sectional endline sample

In the cross-sectional endline sample participants who attended 2+ sessions similarly had a larger variety of play materials (aMD: 0.41; 95% CI: 0.27, 0.55), were more likely to have 1+children’s book (aPD: 0.26; 95% CI: 0.20, 0.33) and were more likely to have heard of lead (aPD: 0.11; 95% CI: 0.06, 0.16) and knew at least one way to avoid harm from lead (aPD: 0.09, 95% CI: 0.05, 0.13) compared with those who attended ≤1 session. Additionally, participants who attended 2+ sessions participated in more play activities (aMD: 0.36; 95% CI: 0.14, 0.57) and had higher nutrition knowledge scores (aMD: 0.22; 95% CI: 0.06, 0.39). Caregiver depressive symptoms were positively associated with session attendance, though CIs for this association crossed the null (table 3).

**Table 3:** Results from adjusted regression analyses in the cross-sectional endline sample (n=1179)

Outcome	Session attendance		Adjusted mean or prevalence difference (95% CI)¶
	2+ (n=337)	≤1 (n=842)	
	Mean±SD or % (n)		
CES-D score	11±8.5	11±8.3	0.32 (-0.97, 1.6)
CES-D score over median (>12)*	32% (109)	29% (247)	0.02 (-0.04, 0.08)
FCI play activities	3.6±1.6	3.3±1.5	0.36 (0.14, 0.57)
FCI play materials	2.3±1.5	1.7±1.3	0.41 (0.27, 0.55)
FCI play activities (4+)	55% (185)	46% (387)	0.08 (0.01, 0.15)
1+ children’s books†	57% (192)	26% (219)	0.26 (0.20, 0.33)
Maternal nutrition knowledge	2.5±1.3	2.3±1.2	0.22 (0.06, 0.39)
Caregiver has heard of lead‡	15% (51)	3.7% (31)	0.11 (0.06, 0.16)
Caregiver knowledge of ways to avoid harm from lead§	9.8% (33)	0.8% (7)	0.09 (0.05, 0.13)

Outcomes: CES-D, CES-D 20-question version (range 0–60, higher scores indicate more depressive symptoms); FCI play activities, FCI play activities subscale (range 0–6) is the total number of play activities that the primary caregiver participated in with the child in the preceding 3 days; FCI play materials, FCI play materials subscale (range 0–6) is the variety of play materials child played with within previous 30 days that were observed in the home; 1+ children’s books indicates the presence of any children’s books in the home; caregiver nutrition knowledge is a sum score of correct responses to six questions about maternal and child nutrition (0–6); caregiver has heard of lead is a binary outcome in response ‘Do you know what lead is?’; caregiver knowledge of ways to avoid harm from lead is a binary outcome that indicates at least one correct response to a question about knowledge of ways to avoid harm from lead.

Mean or prevalence differences in outcomes between the sample that attended 2+sessions, and those that attended ≤1 session. Estimates are from a generalised estimating equation model, adjusted for child age, child sex, maternal and paternal education (completed 5+ years of education), household income (categorical), 4+ antenatal care visits, control over assets, number of children <15 years old (categorical), housing materials (concrete walls and floors) and maternal involvement in the decision-making process.

* Baseline median CES-D score.

† Model not adjusted for father education, income level or floor material due to lack of convergence.

‡ Model not adjusted for father education, income level, number of children or child sex due to lack of convergence.

§ Model only adjusted for child age due to lack of convergence.

¶ n=1,170 for adjusted analyses that adjust for both paternal education (n=2 missing) and income (n=8 missing)

CES-D, Centre for Epidemiologic Studies Depression scale; FCI, family care indicator.

#### Sensitivity analyses

Results using inverse-probability weighting for loss to follow-up and alternative thresholds for session attendance (2+ vs 0 sessions and 1+ vs 0 sessions) were similar to primary results (online supplemental tables 6 and 7). When comparing participants who attended 1–2, 3–5 and 6+ sessions to those who attended 0 sessions, we found that caregivers who attended 3–5 intervention sessions had fewer depressive symptoms than those who did not attend any sessions (aMD: −1.91; 95% CI: −3.69, –0.12) and the households with caregivers who attended 3–5 and 6+ sessions had more play materials (aMD 3–5 vs 0: 0.65, 95% CI: 0.29, 1.00; aMD 6+ vs 0: 0.87, 95% CI: 0.43, 1.30) (online supplemental table 8). The association between caregiver participation in play activities with their child was higher for caregivers who attended 3–5 and 6+ sessions, compared with those who attended 1–2, but these differences were not statistically significant at p<0.05. The involvement of other adult members in play activities with the target child revealed a non-significant trend in the same direction as play activities (online supplemental table 9). When comparing results between participants who reported experiencing more financial loss and food insecurity due to the COVID-19 pandemic to those who had less, we did not observe any consistent trends across analyses (online supplemental figures 1 and 2).

Among participants in the full endline cross-sectional sample, 35% (n=411) had 1+ children’s books in their homes. Among those who attended 2+ sessions, 45% (n=151) reported receiving at least one book from government healthcare workers, while this proportion was 2.5% (n=21) in the comparison group. 21% (n=71) of those who attended 2+sessions and 22% (n=184) of the comparison group bought their child at least one book (online supplemental table 10).

## Discussion

We found that attending two or more group sessions of a multicomponent child development intervention delivered by community health workers in Bangladesh was associated with increases in the availability of a variety of play materials in the home, the prevalence of one or more children’s book in the home and caregiver’s knowledge of lead and ways to avoid harm from lead. These findings were consistent in both the primary longitudinal cohort analysis as well as the supplementary cross-sectional analysis. In the larger cross-sectional sample, we also found that caregiver participation in play activities and knowledge of nutrition were improved among caregivers who attended 2+ sessions, though these associations did not reach statistical significance in the primary cohort sample. Corroboration between findings in the cohort and cross-sectional endline samples for play materials, books in the home, and lead knowledge outcomes adds to our confidence in the primary cohort results. We found preliminary evidence of a dose–response association between the number of group sessions attended and the availability of a variety of play materials and play activities, with more sessions corresponding to larger associations between session attendance and improved outcomes. Given that caregivers who attended sessions were given simple toys and picture books, we are not able to identify if the intervention changed their purchasing behaviour around these items, and instead infer that they successfully received intervention materials that promote early child development.

Attendance at intervention sessions was low throughout the evaluation sample. Of those who attended 2+ sessions in the cohort sample (considered the ‘intervention’ group), most people did not attend more than 4 of the 16 offered sessions. This low attendance likely contributed to smaller intervention impacts on risk factors for poor child development. Compared with the previous intervention that was adapted for this study,^30 31^ the primary cohort analyses from the present study found lower associations between intervention attendance and variety of play materials (MD of 0.58 compared with 1.18), variety of play activities (MD 0.23 compared with 1.05), caregiver depressive symptoms score (MD −0.47 compared with −2.06), awareness of lead (PD of 0.13 compared with 0.52) and awareness to avoid harm from lead (PD of 0.13 compared with 0.54). The previous intervention differed from the present intervention in multiple ways, primary related to delivery. In the previous one, community members were hired to deliver the intervention to one group every 2 weeks, and sessions were delivered in community courtyards close to participants’ homes, as opposed to integrating delivery of multiple sessions a week into the routine duties of health workers, with sessions delivered at community health centres distributed across the subdistrict. Additionally, in the previous intervention, participants were contacted individually prior to each session to promote intervention attendance, which was not done in the present intervention as it was not considered to be a scalable approach. Finally, the previous intervention was delivered to only some villages within a region, as opposed to throughout a study region. In this previous study, intervention attendance was much higher, participants attended on average 78% of 16 offered group sessions.^30^

Two previous responsive caregiving and nutrition interventions that were delivered through community clinics in Bangladesh and measured child development showed larger impacts on similar outcomes, including caregiver depressive symptoms and child stimulation in the home.^26 27^ Session attendance in both previous interventions was substantially higher than we found in the present study. In one study, sessions were delivered to pairs of caregiver–child dyads, and the recruited population attended 76% of the 25 planned sessions, while the evaluation subsample attended 84%.^26^ In the other study, sessions were delivered to groups of four caregiver–child dyads, and mean attendance was 89% of the scheduled 25 sessions.^25^ In addition, neither intervention was delivered at scale in the areas they operated—in both studies, only caregivers of children with low weight-for-age and those who lived a maximum of a 30 min walk from the community-based clinics were recruited. While evaluating an intervention restricted to participants living within a 30 min walk helps to establish the potential for impact, it does not evaluate what the impact would be at the population level if the intervention were to be delivered at scale. Further, a maximum of 24 or 25 caregiver–child dyads per clinic catchment area were recruited to avoid overloading delivery agents.^26^ This approach differs from the present study where all pregnant people and primary caregivers of children under 12 months old living throughout the subdistrict were recruited to participate in the intervention sessions, and there was no restriction on the distance to the community clinic. In addition, group sessions were unrestricted in size and ranged from 10 to 15 caregiver–child dyads compared with two to four in previous work. We hypothesise that differences in observed impacts were due to both higher session attendance and targeting of high-risk children of prior interventions. Early child development interventions that target undernourished children have shown larger impacts on cognitive, language and motor development as well as stimulation in the home and maternal depressive symptoms compared with those that do not specifically target undernourished children.^10^

The present intervention was based on an intervention that was effective when delivered by project staff to a small sample of villages.^30^ We found that the intervention still generated a positive effect, but the magnitude was less when adapted to be delivered by government health workers throughout a geographical region. The results from the present study mirror much of the early child development literature which demonstrates success in small-scale or pilot implementation but less evidence for success at a larger scale,^47^ including recent findings from a monthly home visiting intervention implemented in Pakistan and India which were specifically designed to be feasibly scaled up.^19^ Though the interventions in Pakistan and India had an impact on minimum acceptable diet at 12 months of age, the programmes had no impact on child development outcomes at 18 months or stimulation in the home (including learning materials) at 12 months of age.^19^

Qualitative evaluations during the intervention found that the workload of community health workers was high and may have led to low session quality.^48^ Reducing the burden on health workers by increasing intervention delivery through recorded media or interactive mobile-phone-based contents may be one way to increase quality.^49^ Improving session quality may also lead to increased session attendance over time. Previous work has found that the uptake of community health facility services in Bangladesh is related to service quality (eg, availability and readiness).^50^,^52^ Thus, if session quality were improved, altering the perception of poor health facility quality, caregiver session attendance may improve. In settings where caregivers have access to internet-connected smartphones, mobile-phone-based intervention implementation could also be a feasible and scalable approach and has been previously found to be effective at improving stimulating caregiving practices and caregiver mental health in Zambia and Tanzania.^49^ Additionally, community mobilisation or awareness campaigns may be effective in increasing participation at sessions. These campaigns could work by both increasing the interest and awareness of caregivers as well as that of their family members who may be instrumental in facilitating their participation in sessions.^53^ To improve population-level development outcomes implementation research is required to better understand how to initially reach and sustain the participation of caregivers of young children while implementing high-fidelity interventions in ways that can be scalable through routine systems.

Strengths of this study include the delivery of the intervention throughout a geographic area. The majority of previous interventions evaluated either targeted at-risk populations or were not delivered to all eligible participants. Further, while the study was externally funded and used external trainers, the intervention was delivered by existing government health workers, which is a potentially scalable delivery mechanism. To evaluate the intervention, we leveraged information from a longitudinal cohort to control for outcomes measured at baseline.

The present study has some weaknesses. As the intervention was implemented throughout an entire subdistrict, it could only be evaluated using observational analyses. We were able to leverage the longitudinal data to reduce potential confounding, including employing a difference-in-difference study design and controlling for confounders. However, there may be residual confounding that biased the results. Due to the COVID-19 pandemic, we were only able to deliver 16 out of the originally planned 24 sessions, and the endline survey had to be conducted over the phone, meaning that we were not able to capture the full range of outcomes that were initially planned, including a measure of child development. However, the primary outcome we used has been shown to be correlated with child development outcomes previously in Bangladesh.^42^ A lack of in-person observation may have increased measurement error for questions where an observation component was planned but not conducted since the endline assessment had to be conducted over the phone. For example, the number of books and toys in the home was verified through observation in the baseline survey but only reported over the phone at endline. If participants in the intervention group were more likely to overestimate the number of books and toys in their homes than participants who did not attend sessions, this measurement error could have biased our results. As some of these measures had never been implemented over the phone, we conducted additional training and monitored quality through supervisors joining phone calls to listen to responses. Finally, many of the originally contacted cohort participants were not reachable during the endline phone assessment, and many of the newly recruited participants were not available for a phone interview. We conducted a sensitivity analysis with treatment weights for inverse probability of loss to follow-up to account for this loss in the cohort sample.

## Conclusions

A multicomponent child development intervention delivered to groups of caregivers by government health workers throughout the Chatmohar subdistrict increased the presence of children’s toys and books in the home for families who attended two or more sessions. While group-based interventions have the potential to be more cost-effective and scalable than home visits, the location of intervention sessions, barriers to session attendance, session quality and workload of delivery agents need to be carefully considered in the planning and implementation phases of interventions. Future intervention studies could test the incorporation of alternate delivery locations closer to participants’ homes or prerecorded audio and video materials to improve quality and reduce the burden placed on health workers.

## Supplementary material

10.1136/bmjgh-2024-018736 online supplemental file 1

## Data Availability

Data are available on reasonable request.
