# Supplementary material for: Evaluation of a multicomponent child development intervention delivered through the government health system: a feasibility study
Source: BMJ Glob Health. 2025 Jul 20;10(7):e018736. doi: 10.1136/bmjgh-2024-018736 (PMC12278126; doi:10.1136/bmjgh-2024-018736)
Supplement: online supplemental file 1 [file bmjgh-10-7-s001.docx]

**Evaluation of a multi-component child development intervention delivered through the government health system: A feasibility study**

Jesmin Sultana*, Helen O. Pitchik*, Abul Kasham Shoab, Tarique Md. Nurul Huda, Rezaul Hasan, Fahmida Akter, Tania Jahir, Md. Khobair Hossain, Jyoti B. Das, Ruhul Amin, Farzana Yeasmin, Rizwana Khan, Jenna E. Forsyth, Laura H. Kwong, Jahangir Rashid, Sabina Ashrafee, Mahbubur Rahman, Malay Kanti Mridha, Fahmida Tofail, Peter J. Winch, Stephen P. Luby, Lia C.H. Fernald

*co-first authors

Correspondence to Helen O. Pitchik; [hpitchik@berkeley.edu](mailto:hpitchik@berkeley.edu)

**Supplementary Box 1: Description of multi-stage population proportional sampling at baseline**

| First, a household listing enumerated all pregnant people and caregivers of children under 24 months old. Then, villages with fewer than 25 caregivers of children 6-24 months were merged with the nearest village, so all villages had at least 25 eligible households. After that, 109 villages were selected using probability proportional to size sampling based on the 2011 population census.^27^ Age-stratified random sampling with replacement (if participants refused or were unavailable) was used to select participants within villages (age categories: 6-12 months (n=7), 12-18 months (n=4), and 18-24 months (n=4)). Participants who were 18-24 months at the endline assessment were followed up and part of the longitudinal cohort sample (n=754 followed up, n=517 complete and included in the sample). |
| --- |

| **Supplementary Box 2. Secondary cross-sectional sample methods and results**  *Methods for secondary cross-sectional sample*  As a secondary analysis, we compared differences in outcomes for those exposed to 2+ intervention sessions and ≤1 intervention session among the cross-sectional endline sample. We estimated the differences in outcomes adjusted for confounders using GEEs with standard errors adjusted for clustering by village. We used linear link functions to determine adjusted mean differences for continuous outcomes, and binominal link functions to determine differences in prevalence for binary outcomes. |
| --- |

**Supplementary Table 1: RINEW-G implementation details**

| **Facilitators** | **Group meeting location** | **Major responsibilities beyond intervention** | **Intervention content** | **Group meeting frequency** |
| --- | --- | --- | --- | --- |
| **Pregnant women sessions** | | | | |
| ***Main facilitators*** | |  | Behavioral recommendations on:   - Early childhood stimulation - ANC visits - Maternal nutrition - Maternal mental health - WASH - Lead prevention - Neonatal care and umbilical cord management - Exclusive breastfeeding and colostrum feeding - Kangaroo Mother Care (KMC)   Provision of:   - A food group poster - Kits for Kangaroo Mother Care for low-birth-weight newborns consisting of KMC pouch, socks, and caps - Iron and folic acid supplements for pregnant women | 1 x per month, incorporated with the ANC visits |
| Family welfare visitors | Union Health and Family Welfare Centers,  Satellite Centers | - Counseling for temporary and permanent family planning methods - Provide ANC, PNC services, and counseling for breastfeeding - Senior FWV provides ANC/PNC services at UHC. - Encourage mothers to go to the hospital for a health checkup and delivery - Providing drugs for the children for small problems like common colds, small wound |  |  |
| Midwives | Sub-district Health Complex | - Look after the admitted indoor patients - Follow up with the patients according to the doctor’s advice 24-hour follow-up (usually do 8 hours shifting duty) - Rounds with the doctor and individually at the indoor - Documentation: patient admission, maintain order by the doctor, medical test for diagnosis and treatment - Conduction of normal delivery - Outdoor services: Provide ANC, and PNC (assigned for midwife-trained nurses) - Health education at the outdoor - VIA test for cervical cancer |  |  |
| ***Back-up facilitators*** | |  |  |  |
| Family Welfare Assistant | Community Clinics,  Satellites Centers | - Enrollment and family planning methods for targeted eligible couples - Visit each of the targeted households of the couple within 2 months. - Support FWV during the satellite clinic. - To distribute temporary family planning methods - If they have CSBA training, provide ANC/PNC services to pregnant and lactating mothers - Counseling to stop early marriage |  |  |
| **Mother-baby sessions (for caregivers of children 6-24 months)** | | | | |
| ***Main facilitators*** | |  | Behavioral recommendations on:   - Early childhood stimulation - Maternal and child nutrition - WASH - Lead prevention - Responsive feeding - Thinking healthy   Provision of:   - Age-appropriate toys - Simple picture books - Micronutrient powder for children 6-24 months old - Iron and folic acid supplements for lactating women till the age of 3 months of the child - A food group poster | 2 x per month |
| Community Health Care Providers | Community Clinics | - To look after the patient and provide drugs to the patient from 9am to 3 pm - Provide 28 types of drugs for the patients including iron and folic acid, vitamin, and Calcium for pregnant mother - To give health education at the CC - Extra care for pregnant mothers and 0-5 months children and general patient - To provide all reports on activities from CC using a laptop - To attend monthly meetings at the CC. - Arrange monthly meetings with the Community Group - Arrange meetings every 2 months with Community Support Group |  |  |
| Sub-assistant Community Medical Officer | Union Health and Family Welfare Centers | - 24-hour shifting duty (8 hours shift) - Work in emergency and outdoor - To see all kinds of patients, give prescriptions, and refer them if needed - To do small surgery - Works at union Sub-center and provide Health care facilities |  |  |
| Senior Staff Nurse | Sub-district Health Complex | - Look after the indoor patient - Follow up with the patients according to doctor's advice 24-hour follow-up (usually do 8 hours shifting duty) - Rounds with the doctor and individually at the indoor - Documentation: Patient admission, maintain order by the doctor, medical test for diagnosis and treatment - Conduction of normal delivery at the labor ward - Provide ANC and PNC (assigned for midwife-trained nurses) services at the outdoor - Health education at the outdoor - VIA test for cervical cancer |  |  |
| ***Back-up facilitators*** | | |  |  |
| Health Assistant | Community Clinics,  Satellites Centers | - Invitation and outreach for EPI - EPI activities at the outreach center - Vaccination for all children 0-18 months old - Distribution of de-worming tablets 2 times a year - Health education at the School - Courtyard meeting at the household/village level - To work at the community clinic, including health education - Female HA provides ANC/PNC services - To provide monthly reports of EPI, health education, courtyard meetings, ANC/PNC, and birth and death reports to AHI/HI. AHI and HI then send this report to the statistician and MT-EPI. - Attending monthly field activities review and planning meetings at UHC |  |  |

Notes: WASH: Water, Sanitation and Hygiene; ANC: Antenatal Care;

Kangaroo Mother Care package contained a KMC pouch, socks, and caps

**Supplementary Table 2: Questions used in RINEW-G to assess the caregivers’ nutrition knowledge**

| **SL** | **Question** | **Response** | **Correct answer** |
| --- | --- | --- | --- |
|  | How many extra fistfuls of rice per meal should a woman eat in her 2^nd^ and 3^rd^ trimester? (Ask for a number of fistfuls. If they don’t know, or only know “more than usual”, then mark "don’t know") | ____ fistfuls   1. Don’t know | 1-3 fistfuls of rice |
|  | How many extra fistfuls of rice per meal should a woman eat in the first 6 months after childbirth? (Ask for a number of fistfuls. If they don’t know, or only know “more than usual”, then mark "don’t know") | ____ fistfuls   1. Don’t know | 1-3 fistfuls of rice |
|  | On average, how many Kilograms (Kg) of weight a woman should gain during her entire pregnancy period? | ____ kg   1. Don’t know | Minimum 10 kg weight gain |
|  | How many food groups should a woman eat in a day?  (meat, fish, and poultry is a group, dairy is a group, etc.) | ____ groups   1. Don’t know | At least 5 food groups |
|  | How soon should the baby be put to the breast after birth (in hours)?  If the respondent answers, “immediately”, then write “0” hours | _____ hours   1. Don’t know | Within an hour |
|  | At what age should an infant first receive foods other than breast milk (in months)? | _____ months   1. Don’t know | At 6 months |

**Supplementary Table 3: Socio-demographic characteristics of those followed and lost to follow-up in the longitudinal cohort**

| **Primary Caregiver Characteristics** | **Analytic sample (n=517)** | **Lost to follow-up (n=234)** |
| --- | --- | --- |
|  | **% (n) or mean ± SD** | |
| Age (in years) | 25 ± 5.3 | 26 ± 5.7 |
| Completed primary education (5+ years)^1^ | 80% (416) | 74% (173) |
| Currently pregnant (N=740)^2^ | 1% (6) | 1% (3) |
| Muslim | 98% (507) | 97% (227) |
| **Father Characteristics (N=741)^3^** |  |  |
| Completed primary education (5+ years) | 62% (319) | 56% (128) |
| **Child Characteristics** |  |  |
| Age (in months) | 8.6 ± 1.8 | 8.5 ± 1.9 |
| Child age in Category |  |  |
| < 6 months | 6.6% (34) | 6.0% (14) |
| 6-<14 months | 93% (483) | 94% (220) |
| Female child | 50% (261) | 52% (122) |
| **Household Characteristics** |  |  |
| Household size | 5.2 ± 1.8 | 5.2 ± 1.9 |
| Children <15 years old under care of primary caregiver | 1.9 ± 0.8 | 1.9 ± 0.8 |
| Has cemented floor | 19% (97) | 15% (35) |
| Has brick walls | 23% (121) | 21% (49) |
| **ANC visit status of the caregiver** |  |  |
| Attended at least one visit | 88% (456) | 87% (203) |
| Attended at least four visits | 36% (186) | 29% (67) |
| **Household income (in tertile)** |  |  |
| Low | 50% (259) | 58% (135) |
| Medium | 23% (120) | 18% (43) |
| High | 27% (138) | 24% (56) |
| Caregiver can spend money independently | 56% (292) | 52% (122) |
| Caregiver involved in decision-making process (5+ activities out of 7) | 56% (287) | 62% (146) |

^1^ n=517 for analytic sample; n=233 for lost to follow-up

^2^ n=516 for analytic sample; n=224 for lost to follow-up

^3^ n=512 for analytic sample; n=229 for lost to follow-up

**Supplementary Table 4: Characteristics of the cross-section endline sample**

|  | **Session attendance** | |
| --- | --- | --- |
|  | **2+ (n=337)** | **≤1 (n=842)** |
| **Caregiver Characteristics** | **% (n) or mean ± SD** | |
| Completed primary education (5+ years) | 81% (274) | 85% (714) |
|  | **n=336** | **n=840** |
| Currently pregnant | 2.4% (8) | 2.9% (24) |
| **Father’s characteristics** | **n=335** | **n=842** |
| Completed primary education (5+ years) | 59% (198) | 71% (599) |
| **Child Characteristics** |  |  |
| Age (in months) | 18 ± 5.6 | 17 ± 5.5 |
| 6 - <18months | 49% (166) | 65% (544) |
| 18 - <27 months | 51% (171) | 35% (298) |
| Female | 53% (177) | 48% (404) |
| **Household Characteristics** |  |  |
| Household size | 5.1 ± 1.6 | 5.1 ± 1.8 |
| Children <15 years old under care of primary caregiver | 2.0 ± 1.3 | 1.8 ± 0.8 |
| Has cemented floor | 17% (56) | 24% (203) |
| Has brick walls | 22% (75) | 30% (249) |
| **Antenatal care visits during pregnancy** |  |  |
| Attended at least one visit | 82% (276) | 80% (670) |
| Attended at least four visits | 35% (117) | 33% (277) |
| **Household income (in tertile)** | **n=333** | **n=838** |
| Low | 40% (134) | 39% (315) |
| Medium | 32% (108) | 28% (238) |
| High | 27% (91) | 34% (285) |
| Caregiver can spend money independently | 69% (231) | 64% (535) |
| Caregiver involved in decision-making process (5+ activities out of 7) | 52% (174) | 50% (424) |

**Supplementary Table 5: Intervention session attendance at endline**

|  | **Cohort (n=517)** | **Cross-section (n=1179)** |
| --- | --- | --- |
|  | **Mean ± SD or Median (range) or % (n)** | |
| Mean | 2.6 ± 3.7 | 1.5 ± 2.9 |
| Median (range) | 1 (0-16) | 0 (0-17) |
| **Sessions attended** |  |  |
| 0 | 47% (241) | 60% (702) |
| 1 | 9.3% (48) | 12% (140) |
| 2 | 9.7% (50) | 7.8% (92) |
| 3 | 7.4% (38) | 6.2% (73) |
| 4 | 6.2% (32) | 3.7% (43) |
| 5 | 4.8% (25) | 2.8% (33) |
| 6 | 3.5% (18) | 1.7% (20) |
| 7 | 2.3% (12) | 1.2% (14) |
| ≥8 | 10% (53) | 5.3% (62) |

**Supplementary Table 6: Results from difference-in-differences analysis in the longitudinal cohort (using inverse probability weighting)**

| **Outcome** | **Adjusted DID (95% CI) (IPW)** |
| --- | --- |
| CES-D score | -0.51 (-2.02, 1.01) |
| CES-D score over median (>12)^1^ | -0.03 (-0.13, 0.06) |
| FCI Play activities | 0.25 (-0.10, 0.60) |
| FCI Play materials | 0.58 (0.31, 0.85) |
| FCI Play activities (4+) | 0.05 (-0.06, 0.15) |
| 1+ children’s books^2^ | 0.28 (0.19, 0.36) |
| Maternal nutrition knowledge | 0.08 (-0.16, 0.31) |
| Caregiver has heard of lead^3^ | 0.12 (0.06, 0.18) |
| Caregiver knowledge of ways to avoid harm from lead^4^ | **--** |
| N for analyses adjusted for paternal education: 516, otherwise 517  **Outcomes:** CES-D, Center for Epidemiologic Studies 20-question depression scale (range 0-60, higher scores indicate more depressive symptoms); FCI play activities, Family Care Indicators play activities subscale (range 0-6) is the total number of play activities that the primary caregiver participated in with the child in the preceding three days; FCI play materials, Family Care Indicators play materials subscale (range 0-6) is the variety of play materials child played with within previous 30 days that were observed in the home; 1+ children’s books indicates the presence of any children’s books in the home; caregiver nutrition knowledge is a sum score of correct responses to 6 questions about maternal and child nutrition (0-6); caregiver has heard of lead is a binary outcome in response “Do you know what lead is?”; caregiver knowledge of ways to avoid harm from lead is a binary outcome that indicates at least one correct response to a question about knowledge of ways to avoid harm from lead.  ^1^Baseline median CES-D score  ^2^Adjusted model includes child age and caregiver ANC visits  ^3^Model only adjusted for child age, mother’s education, 4+ ANC visits due to lack of convergence because of small cells  ^4^No adjusted DID estimate is presented for the caregiver’s knowledge of ways to avoid harm from lead because of its low prevalence.  Adjusted DID: Difference-in-Difference estimates from an inverse probability of being followed up weighted generalized estimating equation model, adjusted for child age, child sex, maternal and paternal education (completed 5+ years of education), household income (categorical), 4+ antenatal care visits, control over assets, number of children <15 years old (categorical), housing materials (concrete walls and floors), and maternal involvement in the decision-making process. | |

**Supplementary Table 7: Results from difference-in-differences analysis in the longitudinal cohort (sensitivity analysis comparing 2+ or 1+ sessions to 0 sessions)**

| **Outcome** | **1+ vs 0**  **Adjusted DID (95% CI)** | **2+ vs 0**  **Adjusted DID (95% CI)** |
| --- | --- | --- |
| CES-D score | -0.52 (-2.03, 0.99) | -0.54 (-2.10, 1.02) |
| Had >12 CES-D score^1^ | -0.04 (-0.14, 0.06) | -0.05 (-0.15, 0.06) |
| FCI Play activities | 0.20 (-0.14, 0.54) | 0.24 (-0.12, 0.59) |
| FCI Play materials | 0.52 (0.25, 0.78) | 0.60 (0.32, 0.88) |
| FCI Play activities (mother) (4+) | 0.04 (-0.06, 0.14) | 0.04 (-0.07, 0.15) |
| 1+ children’s books^2^ | 0.22 (0.14, 0.31) | 0.27 (0.18, 0.36) |
| Maternal nutrition knowledge | 0.04 (-0.19, 0.27) | 0.06 (-0.18, 0.31) |
| Caregiver has heard of lead^3^ | 0.11 (0.06, 0.16) | 0.13 (0.07, 0.19) |
| Caregiver knowledge of ways to avoid harm from lead^4^ | **-** | **-** |
| **n’s in each group:** 0=241*; 1+ =276; 2+=228  *240 for adjusted analyses that include paternal education  **Outcomes:** CES-D, Center for Epidemiologic Studies 20-question depression scale (range 0-60, higher scores indicate more depressive symptoms); FCI play activities, Family Care Indicators play activities subscale (range 0-6) is the total number of play activities that the primary caregiver participated in with the child in the preceding three days; FCI play materials, Family Care Indicators play materials subscale (range 0-6) is the variety of play materials child played with within previous 30 days that were observed in the home; 1+ children’s books indicates the presence of any children’s books in the home; caregiver nutrition knowledge is a sum score of correct responses to 6 questions about maternal and child nutrition (0-6); caregiver has heard of lead is a binary outcome in response “Do you know what lead is?”; caregiver knowledge of ways to avoid harm from lead is a binary outcome that indicates at least one correct response to a question about knowledge of ways to avoid harm from lead.  ^1^Baseline median CES-D score  ^2^Adjusted model includes child age and caregiver ANC visits  ^3^Model only adjusted for mother’s education, 4+ ANC visits due to lack of convergence because of small cells  ^4^No adjusted DID estimate is presented for the caregiver’s knowledge of ways to avoid harm from lead because of its low prevalence.  Adjusted DID: Difference-in-Difference estimates (mean differences for continuous outcomes and prevalence differences for binary outcomes) from a generalized estimating equation model adjusted for child age, child sex, maternal and paternal education (completed 5+ years of education), household income (categorical), 4+ antenatal care visits, control over assets, number of children <15 years old (categorical), housing materials (concrete walls and floors), and maternal involvement in the decision-making process. | | |

**Supplementary Table 8: Results from difference-in-differences analysis by categories of session exposure (cohort sample)**

| **Sessions attended** | **n** | **Adjusted difference-in-differences (95% CI)** | | |
| --- | --- | --- | --- | --- |
|  |  | **CES-D score** | **FCI Play activities (mother)** | **FCI Play materials** |
| **0** | 241* | Ref. | Ref. | Ref. |
| **1-2** | 98 | -0.34 (-2.58, 1.89) | -0.16 (-0.63, 0.32) | 0.10 (-0.25, 0.45) |
| **3-5** | 95 | -1.91 (-3.69, -0.12) | 0.40 (-0.03, 0.83) | 0.65 (0.29, 1.00) |
| **6+** | 83 | 0.85 (-1.36, 3.07) | 0.38 (-0.12, 0.89) | 0.87 (0.43, 1.30) |
| Adjusted DID represents Difference-in-Difference estimates from a generalized estimating equation model, adjusted for child age, child sex, maternal and paternal education (completed 5+ years of education), household income (categorical), 4+ antenatal care visits, control over assets, number of children <15 years old (categorical), housing materials (concrete walls and floors), and maternal involvement in the decision-making process.  Analyses stratified by number of sessions attended.  * n=241 for adjusted analyses, 1 participant missing paternal education | | | | |

**Supplementary Table 9: Changes in play activities score by any adult from baseline to endline (cohort sample)**

|  | **Baseline** | | **Endline** | |  |
| --- | --- | --- | --- | --- | --- |
|  | **Sessions attended** | | | |  |
|  | **2+ (N=228)** | **≤1 (N=289)** | **2+ (N=228)** | **≤1 (N=289)** | **Adjusted DID (95% CI)*** |
| **Outcome** | **mean ± SD or % (n)** | | | |  |
| **FCI Play activities 0-6** | 3.0 ± 1.3 | 3.1 ± 1.3 | 4.3 ± 1.4 | 4.0 ± 1.5 | 0.26 (-0.03, 0.56) |
| **FCI Play activities (4+)** | 38% (86) | 37% (106) | 72% (164) | 68% (196) | 0.02 (-0.08, 0.12) |
| Adjusted DID represents Difference-in-Difference estimates from a generalized estimating equation model, adjusted for child age, child sex, maternal and paternal education (completed 5+ years of education), household income (categorical), 4+ antenatal care visits, control over assets, number of children <15 years old (categorical), housing materials (concrete walls and floors), and maternal involvement in the decision-making process  * n=516 for adjusted analyses, 1 participant missing paternal education | | | | | |

**Supplementary Table 10: Sources of children’s book at home among endline cross-sectional samples**

|  | **Session attendance** | |  |
| --- | --- | --- | --- |
|  | **2+ (n=337)** | **≤1 (n=842)** | **Total (n=1179)** |
|  | **% (n) or mean ± SD** | | |
| 1+ child books available at home | 57% (192) | 26% (219) | 35% (411) |
| **Sources (can report multiple)** |  |  |  |
| Bought | 21% (71) | 22% (184) | 22% (255) |
| Govt. health worker | 45% (151) | 2.5% (21) | 15% (172) |
| Elsewhere | 1.8% (6) | 2.5% (21) | 2.3% (27) |

**Supplementary Figure 1: Difference-in-differences plots for CES-D score, FCI play activity score by mother, FCI play material availability at home among groups who had food insecurity due to COVID-19 and who did not have any food insecurity due to COVID-19 at endline.**

**Supplementary Figure 2: Difference-in-differences plots for CES-D score, FCI play activity score with the primary caregiver, FCI play material availability at home among groups who had economic impact due to COVID-19 and who did not have economic impact due to COVID-19 at endline**
